# Development and reliability and validity testing of the college student ecological emotional intelligence scale

**DOI:** 10.3389/fpubh.2026.1807906

**Published:** 2026-05-26

**Authors:** Fei Xu, Wenlong Xu, Chuanchuan Song

**Affiliations:** 1 School of Psychology, Shaanxi Normal University, Xi’an, China; 2 Mental Health Center, Xinyang Normal University, Xinyang, China; 3 School of Educational Science, Xinyang Normal University, Xinyang, China; 4 Mental Health Center, Xinyang University, Xinyang, China

**Keywords:** college students’ emotional intelligence, ecological systems theory, reliability, scale development, validity

## Abstract

**Purpose:**

This study aimed to develop the College Student Ecological Emotional Intelligence Scale and evaluate its reliability and validity.

**Methods:**

Drawing on the three-dimensional structure theory of emotional intelligence and ecological systems theory, we developed an initial 29-item scale through a literature review and expert interviews. Four independent samples were used: Sample 1 (*N* = 5,000) for item analysis and exploratory factor analysis; Sample 2 (*N* = 5,000) for confirmatory factor analysis, criterion-related validity, and reliability testing; Sample 3 (*N* = 18,084) for cross-sample validation and measurement invariance testing; and Sample 4 (*N* = 643) for incremental validity testing after controlling for traditional emotional intelligence.

**Results:**

(1) The final scale contains 29 items across five dimensions: social–emotional regulation ability, ecological mindfulness, negative emotion insight, affective narrative ability, and interpersonal emotional sensitivity. (2) Confirmatory factor analysis supported the five-factor model, with good fit indices (CFI = 0.938, TLI = 0.932, RMSEA = 0.050, SRMR = 0.042). (3) Criterion-related validity analyses showed that total and dimensional scores were positively associated with psychological resilience and negatively associated with SCL-90 symptom factors. (4) Multi-group confirmatory factor analysis supported measurement invariance across gender. (5) The total Cronbach’s alpha coefficient was 0.953, and all subscales met accepted reliability standards.

**Conclusion:**

The College Student Ecological Emotional Intelligence Scale demonstrated sound reliability and validity and may serve as a useful instrument for assessing ecological emotional intelligence among Chinese university students.

## 1 Introduction

Since Salovey and Mayer (1) introduced the construct of emotional intelligence (EI), it has evolved from a specialized psychological concept into a multidisciplinary framework, generating substantial theoretical and practical developments across fields such as education, organizational management, and clinical intervention. Extensive research has shown that emotional intelligence is an important predictor of psychological wellbeing, interpersonal adjustment, and career success (2–8). For college students in emerging adulthood, EI functions as a critical psychological resource for coping with academic and career-related stressors while supporting identity integration and social adaptation. Empirical studies consistently indicate that students with higher EI show clear advantages in managing psychological crises, maintaining high-quality peer relationships, and enhancing subjective wellbeing (7, 9, 10). However, substantial disagreement remains regarding the theoretical models of EI, including competency and hybrid models, as well as their operational definitions (7, 11). This theoretical diversity and the ambiguity that follows directly constrain the precision of assessment tools and their practical usefulness in applied contexts.

A review of major contemporary models of emotional intelligence shows that current research is largely dominated by ability, hybrid, and trait models. Ability models conceptualize EI as a cognitive processing capacity (12), whereas hybrid and trait models incorporate personality and motivational factors (13, 14). When applied to college students, however, these models reveal an important theoretical limitation: they often neglect an ecological interaction perspective. Grounded in Bronfenbrenner’s (15) ecological systems theory, this perspective suggests that emotional intelligence is not simply an intrapsychic trait but a dynamic capacity that develops through ongoing interaction between individuals and their multilayered environments, including microsystems such as peer groups and macrosystems such as cultural values. From this standpoint, emotional competence is inherently situated within specific contexts, such as digital social environments or the academic stressors characteristic of emerging adulthood (16, 17). Understanding EI in college students therefore requires a model that captures how individuals perceive and adapt to diverse environmental cues.

Traditional research has often treated individuals as closed emotional processing systems and assumed that emotional intelligence is a general trait or ability that remains stable across contexts. Ecological systems theory, by contrast, argues that psychological functioning is generated and made meaningful through dynamic interaction with the environment (15). Emotions arise not only from internal physiological responses but also from ecological signals embedded in specific microsystems, such as dormitories and classrooms, and mesosystems, such as teacher-student and peer relationships. Existing models rely heavily on decontextualized assessments and frequently overlook the situational specificity of emotional intelligence (18–21). For example, a student’s competence in regulating internal emotions may not readily transfer to interpersonal situations such as competitive student organizations or critical feedback from faculty members. This context-independent assumption cannot adequately explain why some individuals with high EI scores still struggle with social adaptation in real campus settings. In this sense, ecological EI marks a qualitative shift in the object of intelligence, moving beyond the isolated processing of internal states toward the dynamic interface between the individual and specific environmental stressors, such as devaluation of one’s academic major or challenges associated with social mobility. Within this framework, emotional intelligence is understood not as a fixed internal capacity but as a relational competence enacted through ecological interaction.

To better understand college students’ emotional adaptation, it appears beneficial to move beyond individual-level cognitive or trait perspectives and adopt a dynamic framework centered on person-environment interaction. Such a shift requires expanding the conceptual boundaries of emotional intelligence beyond intrapersonal and interpersonal domains to include the ecological environment. This reconceptualization involves not only redefining the objects of emotional processing but also treating the environment as an independent element within the theoretical structure of EI. Against this background, the three-dimensional structure theory of emotional intelligence offers a useful explanatory framework (22). Rather than relying on a simple dualistic account, this theory proposes a holistic and multidimensional construct that integrates an object dimension (self, interpersonal, and ecological domains), an operational dimension (perception, evaluation, and regulation), and a content dimension (positive versus negative valence). This triadic framework assigns equal theoretical status to ecological emotions alongside self- and interpersonal-oriented emotions, thereby allowing a more nuanced account of emotional functioning in specific campus contexts.

At the measurement level, research on emotional intelligence in college students has relied primarily on general instruments, including the Wong and Law Emotional Intelligence Scale (WLEIS), which is grounded in a trait model; the Mayer-Salovey-Caruso Emotional Intelligence Test (MSCEIT), which reflects a competency model; and the Emotional Quotient Inventory (EQ-i), which is based on a hybrid or trait model (13, 23, 24). Although these instruments have demonstrated acceptable standardization procedures and psychometric properties, their limitations become increasingly evident when they are applied to university student populations.

First, existing measures often lack situational sensitivity. Items in general EI scales are frequently highly abstract. For example, the Wong and Law Emotional Intelligence Scale [WLEIS; (24)] includes items such as “I am sensitive to the feelings and emotions of others.” Such decontextualized wording fails to capture the ecological complexity of college life, which is shaped by nested social systems and context-specific environmental stressors (15). In practice, college students navigate frequent peer interactions, intensive dormitory relationships, and anxieties related to identity development. Here, the ecological character of campus life refers to the functional interdependence between students’ emotional responses and these specific microenvironments. Traditional abstract items do not reflect this interdependence and therefore produce context-blind assessments, reducing predictive validity for the distinctive psychosocial demands of emerging adulthood (20).

Second, the limited ability of existing scales to capture complex emotional processing creates a gap between theoretical assessment and a fuller understanding of psychological adaptation. Emotional intelligence should be conceptualized not only as the capacity to control or recognize emotions but also as the capacity for deeper affective processing. For example, affective narrative ability, that is, the ability to symbolize fragmented experiences, and negative emotion insight, that is, depth of awareness of intrapsychic states, represent higher-order forms of cognitive-emotional integration (25–28). Conventional scales tend to emphasize behavioral self-control and often devote only limited attention to these deeper processing dimensions (26). In university settings, where students face complex challenges related to identity and relationships, incorporating these dimensions into EI assessment allows for a more comprehensive evaluation of adaptive resources.

Furthermore, the development of instruments specifically designed for Chinese university students has lagged behind. Although some domestic studies have adapted foreign scales, these revisions often remain constrained by the original theoretical frameworks and therefore fail to capture the ecological characteristics of local student populations. Within China’s collectivist cultural context, emotional intelligence is often expressed through efforts to maintain balance across multiple relational networks (19, 29). Daily pressures associated with factors such as implicit bias, value devaluation, geographic background, social class, or academic major may require highly specific forms of emotional processing. Existing general instruments are therefore inadequate for such fine-grained ecological assessment.

Against this background, the present study aimed to construct a theoretical framework for College Student Ecological Emotional Intelligence (EEIS) and to develop a psychometrically robust instrument. Drawing on an integrated perspective that combines the three-dimensional structure theory of emotional intelligence with ecological systems theory, we first established a preliminary dimensional framework through literature synthesis and theoretical deduction. We then developed an item pool and subjected it to item analysis and exploratory factor analysis (EFA) to identify its internal factor structure. The resulting model was cross-validated by confirmatory factor analysis (CFA) in an independent sample, followed by multi-group analysis to test measurement invariance across cohorts. Finally, the psychometric properties of the scale were examined through reliability testing and criterion-related validity analysis, using mental health and social adaptation as benchmarks, to ensure its scientific rigor and practical utility.

## 2 Participants and methods

### 2.1 Participants

This study surveyed college students from a university in Henan Province. A total of 29,678 questionnaires were distributed. After excluding 951 invalid questionnaires because of careless responding, incorrect entries, or missing data, 28,727 valid responses were retained, yielding a valid response rate of 96.80%. Participants ranged in age from 18 to 29 years (*M* = 20.55, SD = 1.63), including 9,218 males (32.09%) and 19,509 females (67.91%). To ensure statistical independence and facilitate cross-validation, the total sample was randomly divided into three parts:

Sample 1 included 5,000 randomly selected college students aged 18–25 years (*M* = 20.42, SD = 1.61), including 1,639 males (32.78%) and 3,391 females (67.22%). Sample 2 included 5,000 students randomly drawn from the total sample for confirmatory factor analysis; participants ranged in age from 17 to 29 years (*M* = 20.42, SD = 1.60), including 1,607 males (32.14%) and 3,393 females (67.86%). Sample 3 consisted of 18,084 students and was used for measurement invariance testing and internal consistency analysis; their ages ranged from 17 to 29 years (*M* = 20.43, SD = 1.62), including 5,906 males (32.66%) and 12,178 females (67.34%). Sample 4 (*N* = 643), recruited independently to assess incremental validity, included participants aged 18–25 years (*M* = 20.57, SD = 1.60), including 96 males (14.93%) and 547 females (85.07%). Participants in Sample 4 completed measures of ecological emotional intelligence, traditional trait emotional intelligence (WLEIS), psychological resilience, and the Symptom Self-Rating Scale.

### 2.2 Scale development

The College Student Ecological Emotional Intelligence Scale developed in this study was designed to assess college students’ emotional processing capacities in interaction with the campus ecosystem. Scale development followed standard psychometric procedures and proceeded through the following steps:

First, the theoretical framework of the scale was defined. Based on the three-dimensional structure theory of emotional intelligence [Object-Operation-Content Theory; (22)] and ecological systems theory (15), emotional intelligence was conceptualized as a comprehensive capacity to perceive, express, evaluate, and regulate emotional information concerning the self, others, and the environment within specific ecological contexts. Through an extensive literature review and theoretical analysis, together with observations of college students’ behavior in typical settings such as dormitory interactions, academic competition, and social transitions, we identified five core dimensions for measurement.

Second, to enhance the ecological validity of the initial item pool, we conducted a qualitative phase. Semi-structured interviews were carried out with 16 college students (8 males and 8 females) to explore their lived emotional experiences within the campus ecosystem. Participants were asked to describe specific emotional triggers and regulatory strategies in contexts such as dormitory conflict, academic pressure, and social transition. Interview data were analyzed thematically to identify recurring behavioral indicators of emotional intelligence. These findings, together with the theoretical framework described above, provided the empirical basis for item generation. After the initial items were drafted, content validity was further evaluated. Five psychology experts, including clinical psychology professors and psychometric specialists, reviewed the items for theoretical representativeness, clarity, and cultural relevance. On the basis of their feedback, items that were semantically ambiguous or overlapped with other constructs were revised or removed.

Third, operational definitions were specified for each dimension. Social Emotion Regulation refers to the ability to accurately discern the emotional tone of a group in complex social interactions and, through proactive verbal guidance or behavioral intervention, effectively regulate, elevate, or soothe others’ emotional responses, thereby demonstrating emotional influence and social leadership. Ecological Context Perception refers to heightened sensitivity to emotional cues embedded in broader ecological fields, such as natural environments, social rituals, cultural landscapes, and physical spaces, allowing the individual to detect a setting’s emotional tone and achieve emotional resonance with it. Negative Emotion Insight refers to the ability to perceive, analyze, and reflect on complex negative social phenomena, such as social anxiety or moral conflict, and to understand their underlying psychological significance. Emotional Narrative Ability refers to the capacity to transform ambiguous, fragmented, or highly arousing internal emotional experiences into precise, vivid, and logically organized symbolic language, thereby reconstructing emotional meaning and facilitating effective interpersonal communication. Interpersonal Emotional Sensitivity refers to the individual’s capacity for self-awareness and acute perception of subtle, understated, and potentially negative emotional cues in interpersonal interaction, such as jealousy, belittlement, or covert coldness. This dimension functions as an important psychological mechanism for maintaining close relationships and identifying microtraumatic experiences.

Finally, an initial item pool was constructed and the scoring procedure was established. Based on the operational definitions above, initial items were developed by adapting established items from domestic and international scales, such as the WLEIS and MSCEIT, to the linguistic habits of Chinese university students. Following discussion within a graduate psychology research group and expert review of content validity, semantically ambiguous or redundant items were removed, yielding an initial 90-item scale. The scale uses a 5-point Likert response format ranging from 1 (completely disagree) to 5 (completely agree), with higher scores indicating higher levels of ecological emotional intelligence.

### 2.3 Content validity

The items in this scale were developed through a systematic conceptualization of ecological emotional intelligence and informed by extensive qualitative interview data. After initial item development, five psychology experts, including a clinical psychology professor and psychometric specialists, evaluated the items for representativeness, wording accuracy, and cultural appropriateness. Based on their feedback, ambiguous items were revised and items overlapping with other dimensions were removed. Preliminary administration indicated that participants understood the items clearly. Overall, the scale demonstrated good content validity.

### 2.4 Research instruments

#### 2.4.1 Ecological emotional intelligence scale for college students

The Ecological Emotional Intelligence Scale for College Students (EEIS), developed for this study, was used to assess college students’ ecological emotional abilities. The initial version contained 90 items, each rated on a 5-point scale from strongly disagree to strongly agree. Higher total scores indicate higher levels of ecological emotional intelligence.

#### 2.4.2 Psychological resilience scale

This scale was translated into Chinese and adapted by Xiao Nan and Zhang Jianxin. It uses a 5-point Likert response format and contains 25 items measuring psychological resilience across three dimensions: Self-Mastery (8 items: 1, 5, 7, 8, 9, 10, 24, and 25), Resilience (13 items: 11–23), and Optimism (4 items: 2, 3, 4, and 6). Total scores range from 0 to 125, with higher scores indicating greater psychological resilience (30). In the present study, this instrument demonstrated excellent reliability (Cronbach’s alpha = 0.89).

#### 2.4.3 Symptom self-rating scale

This study used the Chinese revised version of the Symptom Checklist-90 (SCL-90), originally developed by Derogatis et al. (31) and translated by Wang (32). The scale contains 90 items rated on a 5-point scale, where 1 indicates none and 5 indicates severe. It assesses 10 factors: somatization, obsessive-compulsive symptoms, interpersonal sensitivity, depression, anxiety, hostility, phobia, paranoia, psychoticism, and sleep and appetite disturbances. Higher scores on each factor indicate greater severity of the corresponding psychological symptoms.

#### 2.4.4 Wong and Law emotional intelligence scale

The Wong and Law Emotional Intelligence Scale (WLEIS) was administered in Sample 4 as the comparison measure for incremental validity testing. The WLEIS is a widely used self-report instrument assessing trait emotional intelligence. Higher scores indicate higher levels of traditional self-reported emotional intelligence. The scale includes 16 items rated on a 7-point scale from 1 (strongly disagree) to 7 (strongly agree) and covers four domains, including Self Emotional Appraisal, Others’ Emotional Appraisal, Regulation of Emotion, Use of Emotion. In the present study, the scale showed excellent internal consistency (Cronbach’s *α* = 0.91).

### 2.5 Statistical methods

Item analysis, reliability analysis, and criterion-related validity analysis were conducted using SPSS 26.0. Exploratory factor analysis, confirmatory factor analysis, and measurement invariance analysis were performed in R [version 4.3.1; (33)] using the lavaan package [version 0.6–15; (34)].

## 3 Results

### 3.1 Item analysis

The total score was obtained by summing responses across the 90 items in the initial questionnaire. Participants in Sample 1 (*N* = 5,000) were ranked from highest to lowest according to their total scores, with the top 27% designated as the high-score group and the bottom 27% as the low-score group. The critical ratio method and the item-total correlation method were then used to examine item discrimination and validity. Items were retained if they showed a significant difference between the high- and low-score groups (*p* < 0.05), as indicated by the critical ratio (*t-*value), and a corrected item-total correlation (*r-*value) of at least 0.40 (35). As shown in Tables 1, *t-*values ranged from 24.855 to 66.856, and item-total correlations ranged from 0.407 to 0.783, excluding Q1. Although Item 1 (Q1) reached statistical significance (*t* = 24.053, *p* < 0.001), its correlation with the total score was 0.39, which did not meet the retention criterion of 0.40. This finding suggested weak consistency with the overall construct, and the item was therefore removed. The remaining 89 items all showed significant critical ratios (*p* < 0.001) and item-total correlations above 0.40, indicating satisfactory discrimination. Accordingly, 89 items were retained for subsequent exploratory factor analysis.

**Table 1:** Item analysis.

Items	*t*	*r*	Items	*t*	*r*	Items	*t*	*r*	Items	*t*	*r*
Q2	37.813***	0.558***	Q24	33.693***	0.561***	Q47	50.838***	0.701***	Q70	54.426***	0.728***
Q3	36.000***	0.570***	Q25	35.104***	0.579***	Q48	59.205***	0.752***	Q71	56.879***	0.735***
Q4	40.646***	0.610***	Q26	39.835***	0.626***	Q49	56.699***	0.755***	Q72	51.657***	0.701***
Q5	37.810***	0.585***	Q27	41.893***	0.643***	Q50	47.741***	0.651***	Q73	53.193***	0.709***
Q6	43.383***	0.606***	Q28	25.873***	0.407***	Q51	55.942***	0.717***	Q74	49.038***	0.680***
Q7	41.354***	0.618***	Q29	31.466***	0.462***	Q52	48.438***	0.688***	Q75	54.652***	0.679***
Q8	39.273***	0.625***	Q30	42.946***	0.617***	Q53	58.362***	0.718***	Q76	54.016***	0.698***
Q9	39.596***	0.635***	Q31	57.243***	0.726***	Q54	43.820***	0.652***	Q77	45.457***	0.599***
Q10	42.330***	0.649***	Q32	56.147***	0.731***	Q55	52.295***	0.715***	Q78	51.634***	0.711***
Q11	41.936***	0.638***	Q33	63.803***	0.741***	Q56	57.827***	0.738***	Q79	49.659***	0.684***
Q12	35.981***	0.576***	Q34	52.319***	0.727***	Q57	50.599***	0.718***	Q80	55.779***	0.727***
Q13	41.976***	0.638***	Q35	52.337***	0.679***	Q58	48.584***	0.659***	Q81	44.752***	0.597***
Q14	32.017***	0.562***	Q36	58.636***	0.749***	Q59	44.471***	0.660***	Q82	41.201***	0.604***
Q15	39.247***	0.628***	Q37	58.490***	0.728***	Q60	52.362***	0.722***	Q83	53.094***	0.697***
Q16	43.375***	0.651***	Q38	56.243***	0.738***	Q61	52.614***	0.643***	Q84	49.324***	0.693***
Q17	24.855***	0.412***	Q39	46.276***	0.660***	Q62	55.613***	0.690***	Q85	53.946***	0.686***
Q18	34.014***	0.516***	Q40	54.024***	0.722***	Q63	55.866***	0.703***	Q86	53.356***	0.712***
Q19	32.944***	0.540***	Q41	64.118***	0.774***	Q64	45.365***	0.590***	Q87	53.982***	0.703***
Q20	37.285***	0.586***	Q42	57.117***	0.740***	Q65	49.579***	0.689***	Q88	53.980***	0.710***
Q21	42.508***	0.645***	Q43	63.991***	0.773***	Q66	53.105***	0.655***	Q89	43.535***	0.617***
Q22	41.788***	0.642***	Q44	66.856***	0.769***	Q67	55.631***	0.688***	Q90	46.686***	0.647***
Q23	28.044***	0.469***	Q45	65.760***	0.783***	Q68	49.921***	0.702***			
Q24	33.693***	0.561***	Q46	65.900***	0.761***	Q69	58.661***	0.738***			

***p < 0.001.

### 3.2 Factor analysis

#### 3.2.1 Exploratory factor analysis

Exploratory factor analysis was conducted using Sample 1 (*N* = 5,000). The KMO value was 0.965, and Bartlett’s test of sphericity was significant, χ^2^ = 118099.88, df = 406, *p* < 0.001, indicating that the data were suitable for factor analysis. Factors were extracted using principal axis factoring with Promax oblique rotation. Initial factors with eigenvalues greater than 1 were retained, and the final number of factors was determined with reference to the scree plot. During the analysis, items were progressively removed if they met any of the following criteria: (1) an absolute factor loading below 0.50; (2) cross-loadings with a difference of less than 0.20 between two factors; or (3) membership in a factor containing fewer than three items or showing substantial semantic redundancy. Through this iterative process, 60 items were removed because they failed to meet these statistical or theoretical criteria. The final scale contained 29 items loading on five factors and accounted for 67.658% of the total variance. Factor loadings ranged from 0.545 to 0.907, indicating a sound structure. The rotated factor solution is shown in Table 2.

**Table 2:** Exploratory factor analysis results for the ecological emotional intelligence scale (*N* = 5,000).

Indicator	Factor 1		Factor 2		Factor 3		Factor 4		Factor 5	
	Entry	Load	Entry	Load	Entry	Load	Entry	Load	Entry	Load
	Q86	0.907	Q14	0.870	Q32	0.895	Q57	0.837	Q28	0.822
	Q85	0.887	Q9	0.850	Q31	0.816	Q59	0.829	Q29	0.768
	Q87	0.879	Q15	0.847	Q33	0.761	Q55	0.772	Q17	0.577
	Q88	0.867	Q10	0.812	Q36	0.746	Q54	0.757	Q18	0.545
	Q81	0.804	Q8	0.811	Q37	0.722	Q56	0.727		
	Q77	0.790	Q12	0.810	Q38	0.607	Q60	0.709		
	Q75	0.741								
Eigenvalue	5.089		4.527		4.029		3.911		2.055	
Explained variance (%)	17.580		15.612		13.893		13.486		7.087	

Based on the theoretical framework and item content, the five factors were labeled Social Emotion Regulation, Ecological Context Perception, Negative Emotion Insight, Emotional Narrative Ability, and Interpersonal Emotional Sensitivity. Social Emotion Regulation comprises seven items and reflects college students’ capacity to identify group emotional states in social settings and to guide, regulate, elevate, or soothe others’ emotions through verbal and behavioral means. Ecological Context Perception comprises six items and reflects sensitivity to emotional cues in macro-environments, such as natural settings, social rituals, and cultural atmospheres. Negative Emotion Insight comprises six items and reflects the capacity to understand and reflect on complex, negative, and morally charged socioemotional phenomena. Emotional Narrative Ability comprises six items and reflects the capacity to translate abstract and complex inner emotional experiences into precise and vivid verbal expression for effective interpersonal communication. Interpersonal Emotional Sensitivity comprises four items and reflects self-awareness and acute perception of subtle negative social cues in interpersonal interactions, such as being ignored, belittled, or sensing jealousy.

In summary, after exploratory factor analysis, the Ecological Emotional Intelligence Scale was reduced to 29 items.

#### 3.2.2 Confirmatory factor analysis

Confirmatory factor analysis was conducted on Sample 2 (*N* = 5,000) to test the five-factor structure of the EEIS. Analyses were performed in R (version 4.3.1) (33) using the lavaan package (version 0.6–15); (34) with MLR estimation (36). The hypothesized five-factor model showed good fit to the data: χ^2^ = 4944.212, *p* < 0.001; CFI = 0.938; TLI = 0.932; RMSEA = 0.050; and SRMR = 0.042. All fit indices exceeded recommended psychometric thresholds (CFI and TLI > 0.90; RMSEA and SRMR < 0.08). In addition, all 29 items showed substantial standardized loadings on their intended latent factors, ranging from 0.633 to 0.902 (all *p*s < 0.001), indicating that the observed items were strong indicators of the five underlying constructs.

### 3.3 Validity analysis

#### 3.3.1 Construct validity

Construct validity was evaluated through confirmatory factor analysis. As noted above, the five-factor model showed a robust fit in Sample 2 (*N* = 5,000), with all fit indices (CFI = 0.938, TLI = 0.932, RMSEA = 0.050, SRMR = 0.042) meeting or exceeding recommended psychometric standards. All items showed strong standardized loadings on their respective factors, ranging from 0.501 to 0.915 (*p* < 0.001), indicating that the observed variables were good indicators of the underlying latent constructs. This stable factor structure provides statistical support for the theoretical framework of ecological emotional intelligence.

#### 3.3.2 Convergent validity

Convergent validity was assessed using composite reliability (CR) and average variance extracted (AVE). As shown in Table 3, CR values for the five dimensions ranged from 0.785 to 0.946, all exceeding the recommended threshold of 0.70. AVE values for four dimensions—Social Emotion Regulation, Ecological Context Perception, Negative Emotion Insight, and Emotional Narrative Ability—ranged from 0.650 to 0.715, all above the ideal criterion of 0.50. Although the AVE for Interpersonal Emotional Sensitivity was slightly lower (0.478), its CR value (0.785) remained well above 0.70. According to (54), convergent validity can still be considered acceptable when AVE is below 0.50 if CR exceeds 0.60. In addition, McDonald’s omega coefficients for all dimensions (0.786–0.945) further supported strong internal consistency and construct-level convergence.

**Table 3:** Reliability, convergent validity, and discriminant validity of the ecological emotional intelligence scale (*N* = 5,000).

Factor	ω	α	CR	AVE	F1	F2	F3	F4
F1	0.946	0.945	0.946	0.715	–			
F2	0.935	0.934	0.934	0.704	0.317	–		
F3	0.918	0.917	0.918	0.650	0.600	0.615	–	
F4	0.926	0.925	0.926	0.676	0.627	0.657	0.765	–
F5	0.786	0.793	0.785	0.478	0.470	0.355	0.611	0.550
Total	0.956	0.953	–	–	–	–	–	–

*N* = 5,000. Loadings refer to the range of standardized factor loadings (*p* < 0.001). ω, McDonald’s omega; α, Cronbach’s alpha; CR, composite reliability; AVE, average variance extracted. Values below the diagonal are HTMT ratios. Factor names: F1, Social Emotion Regulation; F2, Ecological Context Perception; F3, Negative Emotion Insight; F4, Emotional Narrative Ability; F5, Interpersonal Emotional Sensitivity.

#### 3.3.3 Discriminant validity

Discriminant validity was examined to determine whether the five dimensions represented distinct psychological constructs. We used the heterotrait-monotrait ratio (HTMT), which is generally regarded as a more sensitive and stringent index than the traditional Fornell-Larcker criterion. HTMT values across all pairs of dimensions ranged from 0.317 to 0.765 (53), all below the conservative threshold of 0.85 (37). These findings indicate that, although the dimensions are theoretically related as components of ecological emotional intelligence, they remain sufficiently distinct at the statistical level. Accordingly, the EEIS-29 demonstrated strong discriminant validity, with clear boundaries among its subconstructs (Table 4).

**Table 4:** Criterion-related validity analysis for the ecological emotional intelligence scale.

Factor	Self-strength	Resilience	Optimism	Interpersonal sensitivity	Depression	Anxiety	Overall average score
F1	0.329^***^	0.400^***^	0.289^***^	−0.304^***^	−0.296^***^	−0.254^***^	−0.296^***^
F2	0.319^***^	0.264^***^	0.255^***^	−0.252^***^	−0.284^***^	−0.281^***^	−0.301^***^
F3	0.299^***^	0.300^***^	0.256^***^	−0.224^***^	−0.237^***^	−0.231^***^	−0.253^***^
F4	0.335^***^	0.342^***^	0.276^***^	−0.333^***^	−0.339^***^	−0.308^***^	−0.346^***^
F5	0.144^***^	0.180^***^	0.152^***^	−0.067^***^	−0.069^***^	−0.066^***^	−0.079^***^
Total EEI score	0.372^***^	0.390^***^	0.319^***^	−0.320^***^	−0.329^***^	−0.304^***^	−0.341^***^

^***^p < 0.001. Factor names: F1, Social Emotion Regulation; F2, Ecological Context Perception; F3, Negative Emotion Insight; F4, Emotional Narrative Ability; F5, Interpersonal Emotional Sensitivity.

#### 3.3.4 Criterion-related validity

Using Sample 2 (*N* = 5,000), this study evaluated the criterion-related validity of the Ecological Emotional Intelligence Scale. EEIS total and subscale scores were significantly and positively correlated with all three dimensions of psychological resilience—self-reliance, resilience, and optimism—with coefficients ranging from 0.14 to 0.40 (*p* < 0.001). EEIS scores were also significantly and negatively correlated with all 10 SCL-90 symptom factors and with the total mean SCL-90 score, with coefficients ranging from −0.07 to −0.35 (*p* < 0.01). Notably, the EEIS total score correlated at −0.341 (*p* < 0.001) with the mean total SCL-90 score. These results suggest that higher ecological emotional intelligence is associated with greater psychological resilience and lower levels of psychological symptoms, providing support for the criterion-related validity of the scale.

#### 3.3.5 Incremental validity

To examine incremental validity more rigorously, an additional study was conducted using a new sample (Sample 4, *N* = 643). The purpose was to determine whether the EEIS could explain unique variance in psychological outcomes beyond that explained by existing emotional intelligence measures. Specifically, hierarchical regression analyses were performed to test the predictive value of the EEIS after controlling for scores on the Wong and Law Emotional Intelligence Scale (WLEIS).

As shown in Table 5, the EEIS demonstrated significant incremental validity. For SCL-90 symptoms, adding the EEIS in Model 2 produced a significant increase in explained variance (*R*^2^ = 0.122, *p* < 0.001). Likewise, for psychological resilience, the EEIS explained an additional 9.1% of unique variance (*R*^2^ = 0.091, *p* < 0.001) beyond the WLEIS. In the final models, the EEIS remained a strong and significant predictor of both SCL-90 scores (*β* = −0.349, *p* < 0.001) and resilience (*β* = 0.302, *p* < 0.001), whereas the predictive effect of the WLEIS was not significant. These findings indicate that the ecological perspective on emotional intelligence captures explanatory variance that is not accounted for by traditional EI measures.

**Table 5:** Hierarchical regression results for incremental validity (*N* = 643/412).

Predictors	DV: SCL-90		DV: psychological resilience	
	Model 1 ( β )	Model 2 ( β )	Model 1 ( β )	Model 2 ( β )
WLEIS	0.015	0.002	0.019	0.015
EEIS	–	−0.349^***^	–	0.302^***^
Total *R*^2^	0.000	0.122	0.000	0.091
Adjusted *R*^2^	−0.001	0.119	−0.002	0.087
Delta *R*^2^	–	0.122^***^	–	0.091^***^
F for delta *R*^2^	–	88.74^***^	–	40.91^***^

*p < 0.05, ***p < 0.001. *N* = 643 for the SCL-90 analysis; *N* = 412 for the resilience analysis because of missing data. β represents the standardized regression coefficient in the final step.

### 3.4 Reliability analysis

Reliability was evaluated using Sample 2 (*N* = 5,000). Given the limitations of Cronbach’s alpha, particularly its assumption of essential tau-equivalence (38), both Cronbach’s alpha and McDonald’s omega were calculated to provide a more robust assessment of internal consistency. As shown in Table 3, the total scale demonstrated excellent internal consistency (*α* = 0.953). The Cronbach’s alpha coefficients for the five subdimensions were 0.945 (F1), 0.934 (F2), 0.917 (F3), 0.925 (F4), and 0.783 (F5). McDonald’s omega coefficients ranged from 0.786 to 0.946, consistent with the composite reliability results. All reliability coefficients exceeded the psychometric threshold of 0.70, indicating that the scale provides stable and dependable measurement of ecological emotional intelligence among college students.

### 3.5 Cross-group measurement invariance test

College students are a key population in emotional intelligence research and application. Testing measurement invariance of the College Student Ecological Emotional Intelligence Scale across gender groups is essential to ensure the validity of cross-group comparisons. Using the lavaan package (version 0.6–15) (34), Sample 3 was subjected to sequential tests of configural, metric, scalar, and strict invariance. As shown in Table 6, all models showed acceptable fit. During the sequence of increasingly constrained models, changes in fit indices remained within recommended thresholds, with absolute values of ΔCFI below 0.01 and ΔRMSEA below 0.015. These findings indicate that the scale demonstrated good measurement invariance across male and female groups. Its factor structure, factor loadings, item intercepts, and error variances were essentially equivalent across gender. Accordingly, the scale can be considered comparable across male and female college students, and any observed score differences are more likely to reflect genuine differences in emotional intelligence rather than measurement bias.

**Table 6:** Tests of measurement invariance across gender (*N* = 18,084).

Model	*χ* ^2^	*df*	CFI	TLI	RMSEA	ΔCFI	ΔTLI	ΔRMSEA
Configural invariance	16177.639	734	0.944	0.938	0.060			
Metric invariance	16490.185	758	0.944	0.939	0.059	0.000	0.001	−0.001
Scalar invariance	17188.045	782	0.942	0.939	0.059	−0.002	0.000	0.000
Strict invariance	17669.629	811	0.939	0.939	0.060	−0.003	0.000	0.001

## 4 Discussion

Through qualitative interviews and expert evaluation, this study developed a preliminary Ecological Emotional Intelligence Scale for Chinese university students. After item analysis and exploratory factor analysis, 74 items were selected to form a five-dimensional structure consisting of Social Emotional Regulation Ability, Ecological Context Perception Ability, Negative Emotion Insight Ability, Emotional Narrative Ability, and Interpersonal Emotional Sensitivity. Grounded in an ecological systems perspective, the scale reflects college students’ capacities to perceive, understand, express, and regulate emotions across social, interpersonal, and broader environmental contexts.

The reliability and validity analyses of the College Student Ecological Emotional Intelligence Scale indicate that the instrument has strong psychometric properties. In terms of reliability, the total scale showed a Cronbach’s alpha of 0.953, and both alpha and McDonald’s omega coefficients for each dimension met accepted psychometric standards, indicating high internal consistency and stability. In terms of validity, confirmatory factor analysis showed that the five-factor model fit the data well (CFI = 0.938, TLI = 0.932, RMSEA = 0.050, SRMR = 0.042), supporting the scale’s clear structure and construct validity. Criterion-related validity analyses further showed that total and dimensional scores for ecological emotional intelligence were positively correlated with psychological resilience and negatively correlated with SCL-90 symptom indicators, including depression, anxiety, and interpersonal sensitivity (2, 9). Together, these findings provide strong empirical support for the multidimensional structure of ecological emotional intelligence and its relevance to psychological resources and mental health.

The five dimensions of college students’ ecological emotional intelligence collectively capture the adaptive value of emotion in person-environment interaction. Social Emotional Regulation highlights the capacity to guide emotions within social groups and underscores the importance of emotional leadership and interpersonal harmony in college settings (39–41). Ecological Context Perception, a core feature of the scale, emphasizes the capacity to attune to emotional cues in macro-environments such as natural settings and sociocultural atmospheres, reflecting emotional synchrony between individuals and their surroundings from an ecological perspective (42–44). Negative Emotion Insight reflects the capacity to analyze and reflect on complex negative emotional phenomena and may provide an important basis for psychological resilience and meaning construction (45, 46). Emotional Narrative Ability refers to the capacity to translate ambiguous emotional experience into organized language, a process of symbolization that supports both interpersonal communication and psychological self-reconstruction (26, 47–50). Interpersonal Emotional Sensitivity functions as a microperceptual mechanism through which individuals detect subtle negative social cues, helping identify microtraumatic experiences and preserve the quality of close interpersonal relationships (19, 51, 52). Strengthening college students’ ecological emotional intelligence may therefore enhance social adaptation, reduce psychological stress, and promote broader mental health and social functioning.

Compared with traditional emotional intelligence frameworks that primarily emphasize intrapersonal regulation or interpersonal communication, the ecological emotional intelligence model proposed in this study highlights the dynamic interaction between emotional functioning and environmental contexts. The findings suggest that the EEIS may have practical value for campus mental health services, socioemotional learning, and context-sensitive educational interventions. Specifically, the five dimensions of ecological emotional intelligence may serve as potential targets for peer-support programs, emotional adaptation training, and counseling interventions aimed at improving resilience, emotional reflection, interpersonal functioning, and social adaptation among college students. From an educational perspective, the EEIS may also help universities identify students experiencing difficulties in emotional adaptation within complex campus environments.

Several limitations should be noted. First, the sample was drawn from a single university in Henan Province, which may limit the geographic and ecological generalizability of the findings. Second, the cross-sectional design precludes causal inference and does not allow assessment of the long-term stability of ecological emotional intelligence; future studies should therefore adopt longitudinal designs. Third, although the EEIS showed strong psychometric properties, the study relied exclusively on self-report measures and did not include behavioral outcomes or objective performance indicators. Future research should incorporate multiple methods, such as experience sampling or peer ratings, to further establish the scale’s predictive validity and broader ecological generalizability across sociocultural contexts.

This study identified close associations between ecological emotional intelligence, psychological resilience, and mental health symptoms. Taken together, the findings suggest that ecological emotional intelligence is an important psychological resource for college students as they cope with academic stress, manage interpersonal friction, and maintain mental wellbeing (17). The Ecological Emotional Intelligence Scale developed in this study demonstrated sound psychometric properties and highlighted the positive role of ecological emotional competence in college student development. The study therefore provides a scientifically grounded assessment tool for future localized research on ecological emotional intelligence and for targeted psychological interventions.

## 5 Conclusion

This study developed and validated the College Student Ecological Emotional Intelligence Scale (EEIS) based on ecological systems theory and the three-dimensional structure theory of emotional intelligence. The findings supported a five-factor structure and demonstrated satisfactory reliability and validity. The results further suggest that ecological emotional intelligence is closely associated with psychological resilience and mental health among college students. By emphasizing the dynamic interaction between individuals and their environmental contexts, the EEIS extends traditional conceptualizations of emotional intelligence and provides a culturally relevant assessment tool for future research, psychological assessment, and educational intervention in higher education settings.

## Data Availability

The raw data supporting the conclusions of this article will be made available by the authors, without undue reservation.
